# Diagnosis, Clinical Presentation and Management of Celiac Disease in Children and Adolescents in Poland

**DOI:** 10.3390/jcm13030765

**Published:** 2024-01-29

**Authors:** Joanna B. Bierła, Anna Szaflarska-Popławska, Urszula Grzybowska-Chlebowczyk, Beata Oralewska, Marta Cyba, Grzegorz Oracz, Ewa Konopka, Bożena Cukrowska, Małgorzata Syczewska, Honorata Kołodziejczyk, Petra Rižnik, Jernej Dolinšek

**Affiliations:** 1 Department of Pathomorphology, The Children’s Memorial Health Institute, Aleja Dzieci Polskich 20, 04-730 Warszawa, Poland; e.konopka@ipczd.pl (E.K.); b.cukrowska@ipczd.pl (B.C.); 2 Department of Paediatric Endoscopy and Gastrointestinal Function Testing, Ludwik Rydygier Collegium Medicum in Bydgoszcz, Nicolaus Copernicus University in Torun, Bydgoszcz, 87-100 Toruń, Poland; aszaflarska@wp.pl; 3 Department of Pediatrics, Faculty of Medical Sciences, Medical University of Silesia in Katowice, 40-055 Katowice, Poland; urszulachlebowczyk@wp.pl; 4 Department of Gastroenterology, Hepatology, Nutritional Disorders and Pediatrics, The Children’s Memorial Health Institute, Aleja Dzieci Polskich 20, 04-730 Warsaw, Poland; b.oralewska@ipczd.pl (B.O.); m.cyba@ipczd.pl (M.C.); g.oracz@ipczd.pl (G.O.); 5 Department of Paediatric Rehabilitation, The Children’s Memorial Health Institute, Aleja Dzieci Polskich 20, 04-730 Warszawa, Poland; m.syczewska@ipczd.pl; 6 Laboratory of Anthropology, The Children’s Memorial Health Institute, Aleja Dzieci Polskich 20, 04-730 Warszawa, Poland; h.kolodziejczyk@ipczd.pl; 7 Hepatology and Nutrition Unit, Gastroenterology, Paediatric Department, University Medical Centre Maribor, 2000 Maribor, Slovenia; petra.riznik@gmail.com (P.R.); jernej_dolinsek@hotmail.com (J.D.); 8 Paediatric Department, Faculty of Medicine, University of Maribor, 2000 Maribor, Slovenia

**Keywords:** celiac disease, no-biopsy, risk groups, Polish population

## Abstract

Celiac disease (CD) is a chronic immune-mediated disorder triggered by the ingestion of gluten in genetically predisposed individuals, affecting about 1% of the general population in the developed world. In 2012, the European Society for Pediatric Gastroenterology, Hepatology, and Nutrition (ESPGHAN) recommendations for CD diagnoses in children and adolescents were introduced, allowing the “no-biopsy” approach if certain criteria were met. This approach was also confirmed in the revised guidelines published in 2020. Thus, the aim of this study was to assess—over a one-year period—the clinical presentations and current status of the management of children and adolescents diagnosed with CD in Poland. Medical records of children and adolescents, newly diagnosed with CD in 2022/2023 in three medical centers in Poland, were involved. Gastroenterologists completed the specific anonymous web-based forms developed in the CD SKILLS project, including data routinely assessed at individual visits about the diagnostic approach and clinical presentation of the disease. Our study assessed 100 patients (56% girls) with an age range 1.6–18.0 years. We found that 98% of patients were serologically tested prior to a CD diagnosis and 58% of patients were diagnosed using the “no-biopsy” approach. In the analyzed group, 40% belonged to a known risk group, only 22% had annual screening before the CD diagnosis (the longest for 9 years), and 19% showed no symptoms at the time of the CD diagnosis. Our research confirmed the applicability of the “no-biopsy” approach for the diagnosis of CD in children and adolescents in Poland, and also showed changes in the clinical picture of CD. Moreover, we highlight the need to introduce broad CD serological screening in risk groups of the Polish population.

## 1. Introduction

Coeliac disease (CD) is a chronic immune-mediated disorder triggered by the ingestion of gluten in genetically predisposed individuals, affecting about 1% of the general population in the developed world [1]. In the past, CD was considered a disease that started mainly in childhood, while now we know that CD is a disease that can develop at any age [2,3,4,5]. Moreover, a change in the clinical course of CD toward subclinical and atypical types with dominance of symptoms outside of the gastrointestinal tract has been observed [4,5,6]. In 2012, the European Society for Pediatric Gastroenterology, Hepatology, and Nutrition (ESPGHAN) for the first time introduced recommendations for CD diagnoses in children and adolescents without intestinal biopsy [7]. According to these recommendations, serological tests—screening of tissue transglutaminase 2 antibodies in class IgA (anti-TTG2 IgA) with total IgA—should be the first step in the diagnostic process. The “no-biopsy” approach was introduced in symptomatic patients where the diagnosis was supported when: (a) the concentration of anti-TTG2 IgA in the first blood sample was >10 times the upper limit of normal (>10 × ULN), (b) the second blood sample was positive for endomysial antibodies (EMA IgA), and (c) the presence of human leukocyte antigen (HLA) haplotypes HLA-DQ2 and HLA-DQ8 were confirmed. Furthermore, the experts from ESPGHAN have emphasized the significance of screening for CD in risk groups (including first-degree relatives of CD patients and individuals with comorbidities such as type 1 diabetes mellitus (DMT1), IgA deficiency, autoimmune thyroid disease, Down’s syndrome, Turner syndrome, and Williams syndrome) as a current clinical necessity [8,9]. In 2020, ESPGHAN experts actualized diagnostic guidelines for CD in children and adolescents by recognizing that serological screening is highly effective and safe for the “no-biopsy” diagnosis, and that genetic HLA testing does not improve the sensitivity of the diagnostic approach (and hence can be omitted) [10]. Furthermore, the “no-biopsy” procedure could be conditional, applicable not only to symptomatic patients but also to patients without symptoms, mostly recognized during CD screening of risk groups.

The problem is that despite the extended knowledge of the pathomechanisms of CD development and clear recommendations for CD diagnostics, delays in CD diagnosis in pediatric patients extend (depending on the country) to nearly 3 years. Our previous study of the Polish population showed that the mean duration of symptoms prior to a CD diagnosis in children was significantly shorter than in adults (3.1 and 9 years respectively) [5]. Thus, it is interesting to see how medical centers actually implement the ESPGHAN recommendations for CD diagnostics. It turns out that in 2016 (4 years after the introduction of the ESPGHAN recommendation) in Central Europe only 20.6% of diagnoses were made on the basis of serological tests, and in 2021, this number increased to 63.7% [11,12]. This indicates that implementation of the current ESPGHAN recommendations can take a long time.

Thus, the aim of this study was to assess—over a one-year period—the diagnostic process, clinical presentation, and management of pediatric patients in Poland diagnosed with CD.

## 2. Materials and Methods

### 2.1. Patients

This study was carried out in 2022/2023 as part of the “CD in Focus” project, coordinated within the ESPGHAN Celiac Disease Special Interest Group. It aims to evaluate the management of newly diagnosed patients with CD in different countries across the world.

Medical records of children and adolescents, newly diagnosed with CD in 2022/2023 in 3 medical centers in Poland, were involved. The medical centers were:

- Department of Gastroenterology, Hepatology, Nutrition Disorders, and Pediatrics and the Department of Pathomorphology, The Children’s Memorial Health Institute, Warsaw (POL-1)
- Department of Paediatric Endoscopy and Gastrointestinal Function Testing, Ludwik Rydygier Collegium Medicum in Bydgoszcz, Nicolaus Copernicus University in Torun, Bydgoszcz (POL-2)
- Department of Pediatrics, Faculty of Medical Sciences, Medical University of Silesia in Katowice, Katowice (POL-3)

This study was approved by the local Ethics Committee of the Children’s Memorial Health Institute in Warsaw (19/KBE/2022).

### 2.2. Study Design

A Polish version of an anonymous web-form survey, developed within the CD SKILLS project, was completed by pediatric gastroenterologists. The survey included: (a) the data routinely assessed at the individual visits of newly diagnosed patients (diagnosed prospectively in one of the centers included in the study, in consecutive order) about the diagnostic approach; and (b) the clinical presentation and management of the disease (a sample survey form is given in Supplement S1). None of the surveys collected information containing sensitive patient data, and the surveys were marked with numbers corresponding to the given centers and the order in which the surveys were submitted. The collected data were forwarded to the coordinator by using SurveyMonkey software (Copyright © 1999–2024) (https://www.surveymonkey.com/r/CD-IN-FOCUS-ESPGHAN, accessed on 24 May 2023) and the data were entered into the “CD in Focus” database. Based on the entered data, an Excel file was created, which was then subjected to statistical analysis.

The weight, height, and body mass index (BMI) were analyzed and calculated using https://www.jakicentyl.pl/, accessed on 20 August 2023. This website was created based on percentile charts established during OLA and OLAF research for the Polish population [13]. Moreover, in order to standardize the somatic characteristics of the patients, the following formula was used:

z = (x − µ)/δ

where z is the score of standardization (Z-score); x is the value of the child’s somatic features; µ is the average value of the examined characteristic for children of the same age and sex in the reference population; and δ is the standard deviation for children of the same age and sex in the reference population [14,15,16]. According to the WHO interpretation of anthropometric values, a BMI Z-score of >2 was considered obese, >1 was overweight, and <−2 was malnutrition [17]. Moreover, Z-score values of “weight-for-age” and “height-for-age” below −3 were interpreted as severe wasting and stunting.

Based on the Excel database, the compliance of the laboratory methods used to make the diagnosis with the “no-biopsy” approach was assessed based on the obtained test value after combining it with the cut-off method. Additionally, it was assessed whether the appropriate number of biopsies were taken during endoscopic examinations and whether the assessment/interpretation of the histopathological examination, according to the Marsh–Oberhuber classification [18,19], was consistent with the ESPGHAN recommendations.

### 2.3. Statistical Analysis

Statistical analysis was performed using Statistica 13 (TIBCO Software Inc., StatSoft, Kraków, Poland). Independent samples *t* tests (checking the distribution of parameters in the analysis), Fisher’s exact tests (to compare patients of both sexes and symptoms or lack thereof, for the sum of symptoms—leading and non-leading), Mann–Whitney U tests (to compare the duration of symptoms), and discriminant function analyses were used for the investigation. Discriminant analysis in a model involving the comparison of two groups’ leading symptoms (for example, abdominal pain vs. diarrhea, abdominal pain vs. weight loss, etc.) in terms of the age of the patients at diagnosis and the time from the onset of the first symptoms to the first visit to the gastroenterologist were performed.

## 3. Results

### 3.1. Patients

This study assessed the medical records of 100 patients (56% girls) in whom the diagnosis of CD had been made in three medical centers in Poland (POL-1 *n* = 38; POL-2 *n* = 38; POL-3 *n* = 24). The patients’ characteristics are presented in Table 1. The age of the patients at the time of diagnosis of CD was a median of 8.9 (interquartile range IQR 7.9). In girls, the median age was 7.2 (IQR 7.5) and in the boys, the median age was 9.2 (IQR 8.1). At the time of diagnosis, 96% of patients consumed a normal diet without restrictions, and 4% partially used a gluten-free diet (GFD).

### 3.2. CD Diagnostic Approach

**Figure 1:**
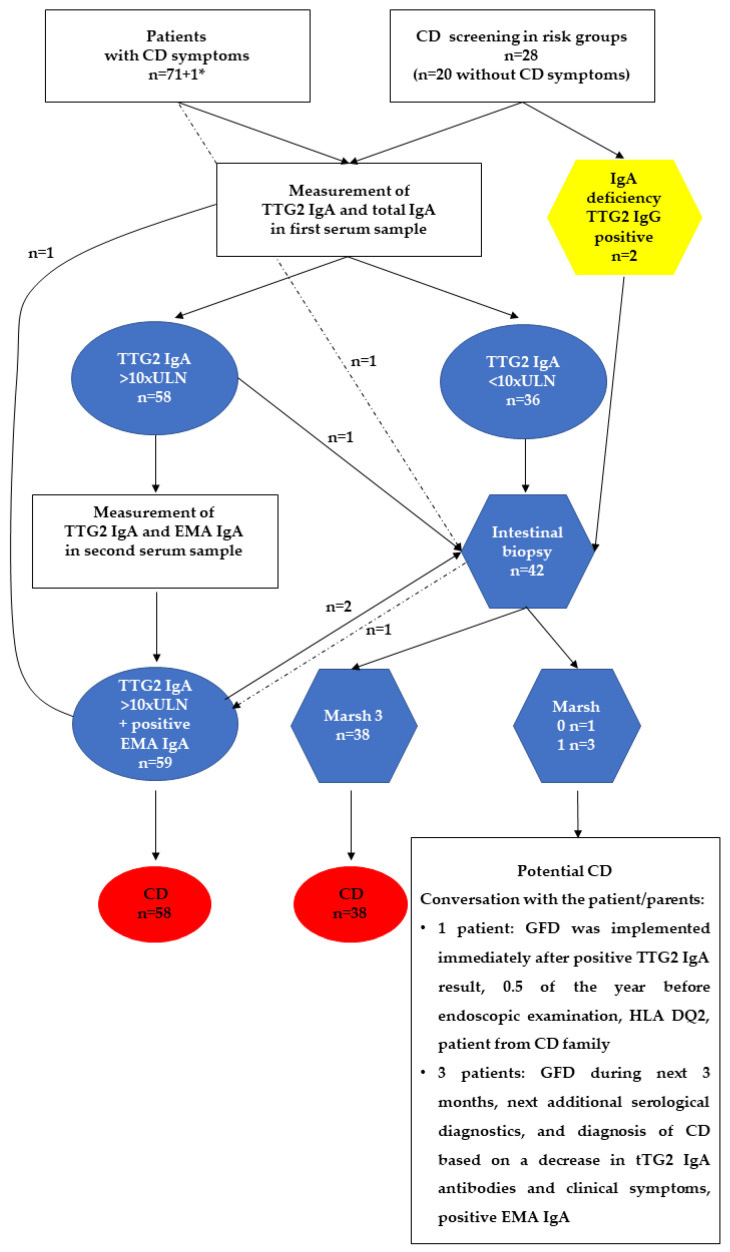
A flow chart showing the CD diagnostic approach in Poland. tTGA2 IgA—tissue transglutaminase 2 antibodies in class IgA; EMA IgA—endomysial antibodies in class IgA; 1*—patient with arthritis. Intestinal biopsy of two children after a parent’s request, and another child had a suspicion of *Helicobacter pylori* gastroduodenitis. The arrow with a dashed line indicates a patient who was diagnosed based on the first intestinal biopsy and a positive serological test result. Marsh 3, 1, or 0 means histopathological examination according to the Marsh–Oberhuber classification [18,19].

Visualization of the CD diagnostic approach in Poland is presented in Figure 1 below.

The analysis showed that 98% of patients were serologically tested prior to a CD diagnosis. The remaining 2% involved two boys. In one of them, intestinal biopsy and histological examination were performed before the serological confirmation of the CD diagnosis, and in the case of the second patient, the parents demanded an intestinal biopsy independently of the serological results. In 58% of patients, the diagnosis was based only on the serological tests: TTG2 IgA and confirmatory EMA-IgA (from the second blood sample). In addition, one patient had a diagnosis based on a single serology sample (TTG2 IgA). For the “no-biopsy” approach, the following tests were used for the measurement of TTG2 IgA: *n* = 23 Phadia100 (chemiluminescence immunoassay, cut-off value 10, Thermo Fisher, Waltham, MA, USA), *n* = 18 BIO-FLASH (chemiluminescence immunoassay, cut-off value 20, Werfen, Bedford, MA, USA), *n* = 10 EUROLINE Coeliac Disease Profile (Western blot, cut-off value 10, EUROIMMUN, Lübeck, Germany), *n* = 4 enzyme immunoassay (ELISA, cut-off value 50, D-Tek, Mons, Belgium) and *n* = 3 not specified test with cut-off value 20. All patients who were diagnosed with CD without a biopsy had TTG2 IgA results that were higher than the ESPGHAN requirement of >10 × ULN.

Intestinal biopsy was performed in 42% of the patients. Most of them (39%) were referred for endoscopy according to the ESPGHAN recommendations (*n* = 34 lower than 10 × ULN or with IgA deficiency and positive TTG2 IgG (*n* = 2)). In two patients with TTG2 IgA higher than 10 × ULN, an intestinal biopsy and histological investigation of the specimens was performed upon parental request (histological changes evaluated as Marsh 3c and Marsh 3b were detected). Additionally, biopsies were performed based on indications such as suspected gastro-duodenal inflammation caused by *Helicobacter pylori* (*n* = 1) or epigastric pain (*n* = 1). The following degrees of histological changes in 42% of patients with CD were found in small intestinal biopsies: Marsh 3a, Marsh 3b, Marsh 3c, and Marsh 1 in 12, 16, 9, and 3 patients, respectively. For patients with a Marsh 1 result, the diagnosis of potential CD was initially made, which was then discussed with the parents/patient. The final diagnosis of CD was made based on a decrease both in symptoms (diarrhea, vomiting, and abdominal pain) and in the concentration of TTG2 IgA antibodies after 3 months of a restrictive GFD, measured by the same test as at the beginning (11-year-old (yo) girl from 385.13 to 110.09 U/mL; 17.9 yo boy 302.97 to 131.00 U/mL; 12 yo girl from 270.7 to 168.0 U/mL, with 50 as the cut-off value of the tests), as well as a positive EMA IgA result. In one case, Marsh 0 was detected; however, this child was from a CD risk group (a first-degree relative in a CD family), with an HLA DQ2 positive result, and was on a GFD introduced by the parents after serological screening, 0.5 year before the histopathological examination.

Statistically significant differences (*p* < 0.01) were found in the duration until a CD diagnosis, the median of which was 1.0 month (IQR 1.8) in patients diagnosed on the basis of serological tests and 3.0 months (IQR 3.8) in patients with a diagnosis based on endoscopy/intestinal biopsies.

### 3.3. CD Screening in At-Risk Groups

Among the patients with CD, 40% (55% girls, *n* = 22) belonged to CD risk groups such as: CD in the family (*n* = 18, 11 girls), DMT1 (*n* = 16, 6 girls), thyroiditis (*n* = 6, 5 girls), IgA deficiency (*n* = 2, 2 girls), Down’s syndrome (*n* = 1), Turner syndrome (*n* = 1), and other: Sjögren syndrome (*n* = 1). Three patients belonged to more than one risk group: a boy (13.6 yo)—Down’s syndrome, thyroiditis, and DMT1; a girl (11.9 yo)—CD in the family, thyroiditis, and DMT1; and a girl (12.6 yo)—IgA deficiency and DMT1.

Characteristics of the serological screening in the CD at-risk groups are presented in Table 2 below. The analysis showed that only 19 (46.34%) of patients from CD risk groups were serologically tested on a regular basis before the CD diagnosis. The longest testing times were 9 years for one patient, 5 years for one patient, 4 years for three patients, 3 years for one patient, 2 years for four patients, and 1 year for nine patients.

Additionally, the following comorbidities were reported: hypogammaglobulinemia (observation for immunodeficiency, one patient), cystic fibrosis (one patient), hypothyroidism (two patients), attention deficit hyperactivity disorder (ADHD, one of them with epilepsy) (two patients), *Helicobacter pylori* gastro-duodenitis (one patient), and three patients with allergies.

### 3.4. CD Symptoms

Diagnostic work-up for CD in 71% of patients was initiated by symptoms. Furthermore, while in 28% of patients (50% were girls) the beginning of CD diagnostics was based on the screening of CD risk groups, only 20% of the study group (42%) were asymptomatic patients. Moreover, one patient (a girl) had arthritis treated with methotrexate for a year without improvement. In this case, only genetic tests and a demonstration of HLA-DQ2 (carried out on the initiative of the parents) directed further proceedings, which led to the diagnosis of CD. There were no statistically significant differences between the age of the patients analyzed depending on gender, symptoms or lack thereof, and belonging to risk groups or not.

Symptomatic patients comprised 79% (*n* = 47 girls), with 25.32% of this group (*n* = 20, 14 girls) being patients in CD risk groups. Their detailed data are included in Table S1, with additional information (not included in this article).

**Figure 2:**
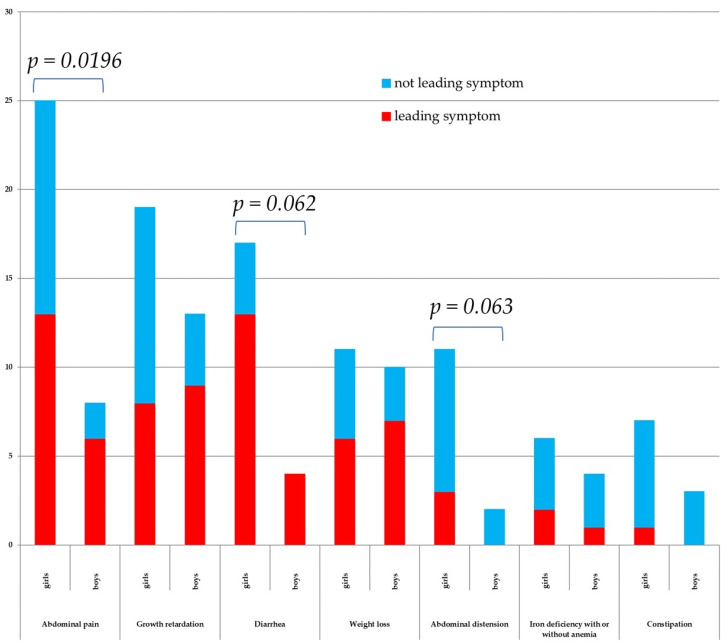
Symptoms reported as initiation of CD diagnostics, divided into the leading and not leading symptoms. Results, whose sum exceeds 5%, are presented as % of total number of patients with CD. Fisher’s exact test statistic values for the sum of symptoms (leading and non-leading) are presented in cases of statistically significant or tendency results.

Patients reported their leading symptoms as abdominal pain (19% of the total number of patients with CD), growth retardation (17%), diarrhea (17%), weight loss (13%), abdominal distension (3%), iron deficiency (3%), *Dermatitis herpetiformis* Duhring (DHD) (3%), constipation (1%), and unexplained fatigue, unexplained irritability, and flatulence (Figure 2). The survey also contained questions about dental enamel defects and ataxia, but none of the patients reported such symptoms. Among 40 patients (*n* = 22 girls) from the described CD risk groups, symptoms were present in 20 patients (50% of the CD risk group, *n* = 14 girls). In this group, the leading symptoms were abdominal pain (*n* = 5, four girls) and weight loss (*n* = 5, two girls), and in addition, diarrhea (*n* = 4, only girls), growth retardation (*n* = 3, two girls), abdominal distension (*n* = 2, only girls), and iron deficiency (*n* = 1).

Statistical analysis of the symptomatic patients, depending on gender and a specific symptom, showed that the sum of symptoms (leading and non-leading), such as abdominal pain, was significantly higher in girls (Figure 2). Moreover, in girls, the sum of symptoms (leading and non-leading), such as abdominal distension and diarrhea, had a tendency to predominate, but this value was not statistically significant.

The majority of symptomatic children (43.0%, *n* = 34) were monosymptomatic, followed by patients with two (30.4%, *n* = 24), three (17.7%, *n* = 14), four (5.1%, *n* = 4), or five (3.8%, *n* = 3) symptoms. Among monosymptomatic children thirteen patients had abdominal pain, seven patients had growth retardation, four patients had diarrhea or weight loss, two patients had DHD or iron deficiency, and one patient had unexplained fatigue or unexplained irritability.

The duration of symptoms before the first visit to the gastroenterologist was a median of 0.5 (IQR 1.74) years, with the longest being 10.92 years (in a case of growth retardation). The average time values calculated for the leading symptoms (occurring in a group of at least three patients) were: for abdominal pain, a median of 0.50 years (IQR 0.75); for weight loss, a median of 0.25 years (IQR 0.84); for diarrhea, a median of 0.46 years (IQR 0.79); for growth retardation, a median of 0.79 years (IQR 5.42); for DHD, a median of 2.0 years (IQR 4.42); and for iron deficiency/anemia, a median of 1.0 year (IQR 4.48). Discriminant analysis showed statistically significant differences in the duration of symptoms between patients with leading symptoms of growth retardation vs. abdominal pain (*p* = 0.032) or diarrhea (*p* = 0.036).

Statistical analysis of the leading symptoms and the time from the onset of the first symptoms to the first visit to a gastroenterologist showed that only for diarrhea was there a statistically significant difference by the gender of the patients. In girls, this interval was statistically significantly (*p* = 0.008) shorter and averaged 0.25 years (IQR 0.34) compared to 2.2 years (IQR 4.41) in boys.

The BMI Z-score at the time of diagnosis of CD was a median of −0.66 (IQR 1.57). In girls, there was a median BMI Z-score of −0.64 (IQR 1.53), and in boys, a median BMI Z-score of −0.82 (IQR 1.53). A BMI Z score < −2 (malnutrition) was detected in four of the patients with CD (*n* = 2, both girls). Furthermore, nine of the patients with CD were overweight (*n* = 5, all girls) and two of the patients (only boys) were considered obese.

The Z-score values for “height-for-age” for all patients with CD was a median of −0.87 (IQR 2.31). For girls, it was a median of −0.81 (IQR 2.42), and for boys, it was a median of −0.94 (IQR 1.99). The analysis of Z-score values of “height-for-age” showed that the results of three boys (two of them overweight) with CD should be interpreted as severe stunting, but they were classified by a gastroenterologist as asymptomatic.

The Z-score values for “weight-for-age” for all patients with CD was a median of −0.32 (IQR 1.00). For girls, there was a median of −0.28 (IQR 1.08) and for boys, there was a median of −0.45 (IQR 0.97). Wasting was not detected among patients with CD according to the analysis of Z-score values for “weight-for-age.”

### 3.5. CD Management

**Figure 3:**
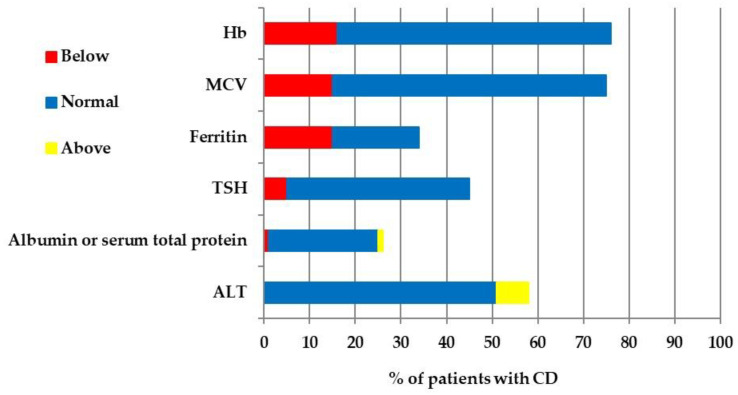
Results of additional blood checkup. Hb—hemoglobin, MCV—mean corpuscular volume, TSH—thyroid-stimulating hormone, ALT—alanine aminotransferase.

Additionally, 76% of patients had a blood checkup and the percentage of patients with normal, below, and above normal results is presented in Figure 3 below. The survey included a question about checkups of bone mineral density, but this test was not performed in any patient.

The final diagnosis of CD was communicated to the patient/family during “face to face” visits in 57% of cases, by a telephone call in 33% of cases, and by a written letter in 10% of patients. The first follow-up clinic visit was then scheduled. The first appointment after the final CD diagnosis was scheduled after one or two months (5% of patients with CD), after three months (47% of patients), after four months (7% of patients), after five or eight months (2% of patients), after six months (24% of patients), or after seven or nine months (1% of patients).

Moreover, during communication of the CD diagnosis, 65% of patients were advised to join the local/national celiac society, whilst for 33% of patients it is not known whether such information was provided. The results for 2% of patients were not remitted because one patient will remain under the care of another center and the second patient belongs to a society of patients with DMT1.

## 4. Discussion

Our research is the first attempt to describe the actual introduction of the new ESPGHAN CD diagnostic recommendations in Poland. It needs to be highlighted that in our study, 98% of CD diagnostic procedures started with serological screening. Implementation of the new recommendations can improve the well-being of patients because 56% of CD diagnoses were made based on the “no-biopsy” approach. Other researchers have also shown that the use of the “no-biopsy” approach has been increasing in European countries in the last few years [12]. The introduction of the “no-biopsy” approach not only has a huge impact on the well-being of patients but can be considered a serious economic factor for research settlements. Patients in this group did not undergo the expensive endoscopic procedure, often combined with general anesthesia. Only in two patients did the parents require an endoscopic procedure. This shows an increase in confidence in the reliability of laboratory tests.

Particular attention should be paid to ensuring that the time between the serological test and the histological test is not too long. In the case of one of our patients, the parents independently implemented a GFD for half a year before the endoscopic examination, which questions the validity of such an examination and may constitute a basis for questioning the diagnosis of CD. Therefore, on the one hand, the diagnosis of CD in patients with Marsh 0/1 presented in this study can be considered inconsistent with the ESPGHAN guidelines. However, for the sake of the patient’s well-being, after an individual conversation with the parents/patient, observing the reduction in symptoms and tTG2 IgA concentration on a gluten-free diet and the positive EMA IgA, a high probability of CD should be considered. Additionally, one CD diagnosis made on the basis of a single blood sample, inconsistent with ESPGHAN recommendations, raises doubts.

We found a significant increase in the median age of children at CD diagnosis [6,20,21,22,23]. Moreover, children in Poland are nearly 2 years older at CD diagnosis, compared to data obtained in studies in Croatia, Germany, Hungary, Italy, and Slovenia [23]. Similar to previous studies, girls dominated our research group [22,23,24].

Among the CD diagnoses, more than one-third of patients came from risk groups. However, only 46.3% of patients from risk groups were tested, which indicates the need for extensive information campaigns among both doctors and patients in Poland. CD screening was mainly performed in first-degree relatives and DMT1. “CD in the family” was declared in 18% of all patients, which is similar to previous studies [23]. Of the comorbidities, the largest group was patients with DMT1, and the second largest group was those with thyroiditis, which is the opposite of the studies obtained by Riznik [23]. This may be related to the extensive research on patients with DMT1 conducted in POL-1.

As in the previous study by Riznik [23], the leading symptoms of patients with CD were abdominal pain, growth retardation, and diarrhea. With regard to gender, it is worth noting that girls had statistically significantly more frequent symptoms such as abdominal pain, diarrhea, and abdominal distension. Moreover, it was noticed that when the dominant symptom was diarrhea, girls were referred to gastroenterologist care at a statistically significantly faster rate (median 0.25 years vs. 2.21 years) compared to boys.

Statistically, the longest time it took for patient referral to a gastroenterologist was when the patient’s main symptom was growth retardation. This time was statistically significantly longer, especially in comparison to abdominal pain or diarrhea. Moreover, three boys were classified as asymptomatic patients, even though their Z-score values of “height-for-age” should be interpreted as severe stunting.

Our study showed that 24% of patients did not have any additional tests performed at the time of diagnosis of CD, which is inconsistent with the ESPGHAN recommendations [25]. This is very disturbing because 22.37% of the tested patients showed iron deficiency with or without anemia. Maintaining this percentage proportion, hypothetically in the group of untested patients (*n* = 24), five of them could have anemia. The appropriate supplementation was not implemented in these patients, which may hinder their recovery process on a GFD.

Total protein and serum albumin levels were not tested in 75% of patients with CD at the time of CD diagnosis. Recent data showed that the determination of serum albumin and prealbumin should not serve as proxy measures of total body protein or total muscle mass, and should not be used as nutrition markers [26]. However, a decreased concentration of visceral proteins, particularly serum albumin, during the acute phase response is a good marker of increased tissue catabolism. Moreover, albumin is a key extracellular antioxidant and serves as a ligand for pro-oxidative metals, such as copper and iron [27]. Therefore, it is worth considering testing for albumin and total protein levels in patients with CD, with a particular indication in patients with CD with anemia.

It is worth noting that 7% of patients diagnosed with CD had liver enzyme activity elevated above the norm, while 42% of patients did not have such tests performed. It is unknown what the outcomes were after GFD implementation. However, it is worth considering the implementation of these analyses in the diagnosis of CD patients on a GFD, because abnormal liver function may be the reason for prolonged convalescence of the patient. Moreover, it is worth considering a diagnosis of CD in patients with liver diseases, and not only of autoimmune origin, e.g., autoimmune hepatitis. In our previous study of patients with Wilson’s disease, CD was detected in 2.7% of patients [28].

From our point of view, the main barriers to the implementation of the ESPGHAN CD recommendations in a given country are the availability of information on changes in the native language for both gastroenterologists and pediatricians, as well as administrative guidelines, that determine the financing and availability of diagnostic methods. Such a high percentage of the share of serological tests in the diagnosis of CD, presented in this article, is greatly influenced by the involvement of the co-authors in the functioning of the Coeliac Section of the Polish Society of Pediatric Gastroenterology, Hepatology, and Nutrition. The main goal of this section is to disseminate knowledge in the field of diagnosis and treatment of CD and other gluten-dependent diseases; to improve the scientific knowledge and expertise of doctors of various specialties, laboratory diagnosticians, and dietitians; as well as to cooperate with patient communities and support their activities [29]. Additionally, the co-authors of this publication, who are also members of ESPGHAN, are involved in publishing reports on the latest information in Polish national journals, e.g., after annual conferences [30,31,32]. However, it is worth paying attention to one fact—even the best recommendations and the willingness of people involved in their dissemination will not change anything if there is no administratively established financing of diagnostic tests. For example, in 2017, in accordance with the regulation of the Polish Minister of Health, the National Health Fund financed the following antibody tests (settled as part of the consultation) as a guaranteed service for both children and adults: anti-endomysium, anti-smooth muscle cell, anti-gliadin IgG class, and anti-gliadin IgA class [33]. From 1 October 2022, the rules for financing research have changed and referrals for serological screening (TTG2 IgA) can be issued by medical specialists (e.g., gastroenterologists or orthopedists) [34]. Unfortunately, this group did not include pediatricians or family physicians. This administrative decision therefore bypassed doctors who could refer patients from risk groups without symptoms of CD (especially first-degree relatives) for such tests every year. Our study shows that such diagnostics are extremely important in Poland and efforts should be made to expand them.

### Study Limitation

The main limitation of this study concerns the number of included research centers and their degree of reference. Only three centers from Poland, with a third degree of reference, employing gastroenterologists with high competencies, were included in this study.

## 5. Conclusions

Our research confirmed that the new recommendations for CD diagnoses in children and adolescents were successfully introduced in Poland, especially the introduction of the “no-biopsy” procedure. Additionally, the change in the clinical picture of CD was confirmed. Moreover, we highlight the need to introduce broad CD serological screening in risk groups of the Polish population.

## Figures and Tables

**Table 1:** Patients’ characteristics.

	Girls *n* or Range	Boys *n* or Range
Number of patients	56	44
Age (years)	1.6–18.0	3.4–18.0
Growth (cm)	58–173	92–177
Body weight (kg)	11–57	12–70
BMI	12.65–32.70	11.42–26.87
CD risk groups	22	18
Asymptomatic	8	11
“No-biopsy” approach	30	28
Endoscopy/intestinal biopsies	26 Marsh 3 (*n* = 1 Marsh 0; *n* = 2 Marsh 1)	16 Marsh 3 (*n* = 1 Marsh 1)

*n*—number of patients; BMI—body mass index. “No-biopsy” approach—the diagnosis of these patients with CD was based solely on serological tests. Endoscopy/intestinal biopsies—the diagnosis of these patients with CD was based solely on serological tests and histopathological examination, with the use of the Marsh–Oberhuber classification [18,19].

**Table 2:** Patients in CD risk groups.

	CD Risk Groups						
	CD in Family	IgA Deficiency	Thyroiditis	DMT1	Down’s Syndrome	Turner Syndrome	Other:
Since birth:	7	0	0	0	1	1	1
<1 year:	0	0	1	9	0		
1–5 years:	8	2	4	5	0		
5–10 years:	3	0	1	2	0		
SUM = 46 *	18	2	6	16	1	1	1
Tested (% of risk group) **	10 (55.56%)	1 (50.00%)	2 (33.33%)	7 (43.75%)	1 (100%)	1 (100%)	0 (0%)

DMT1—diabetes mellitus type 1; other: Sjögren syndrome. * In this group, three patients belonged to more than one CD risk group: a boy (13.6 years old, yo)—Down’s syndrome, thyroiditis, and DMT1; a girl (11.9 yo)—CD in the family, thyroiditis, and DMT1; a girl (12.6 yo)—IgA deficiency and DMT1. ** Percentage of results for the question: has a patient belonging to a known risk group been serologically tested for CD on a regular basis before the diagnosis?

## Data Availability

The data are available upon request.

## Supplementary Materials

The following supporting information can be downloaded at: https://www.mdpi.com/article/10.3390/jcm13030765/s1, sample survey form in Supplement S1; Table S1: (List of symptomatic patients (*n* = 79, 47 girls), including the division into leading and non-leading symptoms, gender, and CD risk groups).

## References

[1] Lundin K.E.A., Qiao S.W., Snir O., Sollid L.M. (2015). Coeliac disease—From genetic and immunological studies to clinical applications. Scand. J. Gastroenterol..

[2] Lurie Y., Landau D.A., Pfeffer J., Oren R. (2008). Celiac disease diagnosed in the elderly. J. Clin. Gastroenterol..

[3] Husby S., Murray J.A. (2014). Diagnosing coeliac disease and the potential for serological markers. Nat. Rev. Gastroenterol. Hepatol..

[4] Majsiak E., Choina M., Golicki D., Gray A.M., Cukrowska B. (2021). The impact of symptoms on quality of life before and after diagnosis of coeliac disease: The results from a Polish population survey and comparison with the results from the United Kingdom. BMC Gastroenterol..

[5] Majsiak E., Choina M., Gray A.M., Wysokiński M., Cukrowska B. (2022). Clinical manifestation and diagnostic process of celiac disease in Poland—Comparison of pediatric and adult patients in retrospective study. Nutrients.

[6] Rybak A., Socha P., Stolarczyk A., Cukrowska B., Obrycki Ł., Wierzbicka A., Oralewska B., Szaflarska-Popławska A., Iwańczak B., Jarocka-Cyrta E. (2014). Clinical picture of celiac disease in children in Poland. Standardy Med. Pediatria.

[7] Husby S., Koletzko S., Korponay-Szabó I.R., Mearin M.L., Phillips A., Shamir R., Troncone R., Giersiepen K., Branski D., Catassi C. (2012). European Society for Pediatric Gastroenterology, Hepatology, and Nutrition guidelines for the diagnosis of coeliac disease. J. Pediatr. Gastroenterol. Nutr..

[8] Ludvigsson J.F., Card T.R., Kaukinen K., Bai J., Zingone F., Sanders D.S., Murray J.A. (2015). Screening for celiac disease in the general population and in high-risk groups. United Eur. Gastroenterol. J..

[9] Parzanese I., Qehajaj D., Patrinicola F., Aralica M., Chiriva-Internati M., Stifter S., Elli L., Grizzi F. (2017). Celiac disease: From pathophysiology to treatment. World J. Gastrointest. Pathophysiol..

[10] Husby S., Koletzko S., Korponay-Szabó I., Kurppa K., Mearin M.L., Ribes-Koninckx C., Shamir R., Troncone R., Auricchio R., Castillejo G. (2020). European Society Paediatric Gastroenterology, Hepatology and Nutrition Guidelines for Diagnosing Coeliac Disease. J. Pediatr. Gastroenterol. Nutr..

[11] Riznik P., De Leo L., Dolinsek J., Gyimesi J., Klemenak M., Koletzko B., Koletzko S., Korponay-Szabó I.R., Krencnik T., Not T. (2019). Diagnostic Delays in Children with Coeliac Disease in the Central European Region. J. Pediatr. Gastroenterol. Nutr..

[12] Riznik P., Carnohorski I., Dolinsek J., Dragutinovic N., Gyimesi J., Hauer A.H., Kamhi Trop T., Klemenak M., Korponay-Szabo I.R., Krencnik T. (2023). Diagnostic approach in children with newly diagnosed coeliac disease—Are the ESPGHAN guidelines followed appropriately? In Proceedings of the ESPGHAN 55th Annual Meeting, Vienna, Austria, 17–20 May 2023. J. Pediatr. Gastroenterol. Nutr..

[13] Serwis JAKICENTYL.PL. https://www.jakicentyl.pl/.

[14] Kułaga Z., Różdżyńska A., Palczewska A., Grajda A., Gurzkowska B., Napieralska E., Litwin M. (2010). Siatki centylowe wysokości, masy ciała i wskaźnika masy ciała dzieci i młodzieży w Polsce—Wyniki badania OLAF. Stand. Med. Pediatr..

[15] Kułaga Z., Litwin M., Tkaczyk M., Palczewska I., Zajączkowska M., Zwolińska D., Krynicki T., Wasilewska A., Moczulska A., Morawiec-Knysak A. (2011). Polish 2010 growth references for school-aged children and adolescents. Eur. J. Pediatr..

[16] Kułaga Z., Grajda A., Gurzkowska B., Góźdź M., Wojtyło M., Swiąder A., Różdżyńska-Świątkowska A., Litwin M. (2013). Polish 2012 growth references for preschool children. Eur. J. Pediatr..

[17] de Onis M., Blössner M. (2003). The World Health Organization Global Database on Child Growth and Malnutrition: Methodology and applications. Int. J. Epidemiol..

[18] Marsh M.N. (1992). Gluten, major histocompatibility complex and the small intestine: A molecular and immunobiologic approach to the spectrum of gluten sensitivity (celiac sprue). Gastroenterology.

[19] Oberhuber G., Granditsch G., Vogelsang H. (1999). The histopathology of coeliac disease time for a standardized report scheme for pathologists. Eur. J. Gastroenterol. Hepatol..

[20] Ravikumara M., Tuthill D.P., Jenkins H.R. (2006). The changing clinical presentation of coeliac disease. Arch. Dis. Child..

[21] Tapsas D., Holle´n E., Stenhammar L., Fälth-Magnusson K. (2016). The clinical presentation of coeliac disease in 1030 Swedish children: Changing features over the past four decades. Dig. Liver Dis..

[22] Kivelä L., Kaukinen K., Lähdeaho M.L., Huhtala H., Ashorn M., Ruuska T., Hiltunen P., Visakorpi J., Mäki M., Kurppa K. (2015). Presentation of celiac disease in Finnish children is no longer changing: A 50-year perspective. J. Pediatr..

[23] Riznik P., De Leo L., Dolinsek J., Gyimesi J., Klemenak M., Koletzko B., Koletzko S., Korponay-Szabó I.R., Krencnik T., Not T. (2021). Clinical Presentation in Children with Coeliac Disease in Central Europe. J. Pediatr. Gastroenterol. Nutr..

[24] Van Kalleveen M.W., De Meij T., Plötz F.B. (2018). Clinical spectrum of paediatric coeliac disease: A 10-year single-centre experience. Eur. J. Pediatr..

[25] Mearin M.L., Agardh D., Antunes H., Al-Toma A., Auricchio R., Castillejo G., Catassi C., Ciacci C., Discepolo V., Dolinsek J. (2022). Position Paper on Management and Follow-up of Children and Adolescents With Celiac Disease. J. Pediatr. Gastroenterol. Nutr..

[26] Evans D.C., Corkins M.R., Malone A., Miller S., Mogensen K.M., Guenter P., Jensen G.L., ASPEN Malnutrition Committee (2021). The Use of Visceral Proteins as Nutrition Markers: An ASPEN Position Paper. Nutr. Clin. Pract..

[27] Soeters P.B., Wolfe R.R., Shenkin A. (2019). Hypoalbuminemia: Pathogenesis and clinical significance. J. Parenter. Enteral. Nutr..

[28] Jańczyk W., Bierła J.B., Trojanowska I., Wierzbicka-Rucińska A., Cukrowska B., Socha P. (2023). Prevalence and significance of autoantibody seropositivity in children with Wilson’s disease. Diagnostics.

[29] Coeliac Section of the Polish Society of Pediatric Gastroenterology, Hepatology and Nutrition. https://ptghizd.pl/sekcje/sekcja-celiakalna/.

[30] Bierła J.B., Cukrowska B. (2018). News about celiac disease—Report from the celiac session of the 51st congress ESPGHAN 2018. Stand. Med. Pediatr..

[31] Bierła J.B., Majsiak E., Cukrowska B. (2019). Report of the 18th International Celiac Disease Symposium. Stand. Med. Pediatr..

[32] Bierła J.B. (2022). Celiac disease news—Guidelines from the 54th annual meeting of ESPGHAN (2022). Stand. Med. Pediatr..

[33] Krzyżanowska-Sołtysiak M. (2017). Tests to Detect Celiac Disease Are Free in Poland. https://bezglutenowamama.pl/badania-wykrywajace-celiakie-sa-w-polsce-bezplatne.

[34] Krzyżanowska-Sołtysiak M. (2022). Can a Family Doctor Order Blood Tests to Detect Celiac Disease?. https://bezglutenowamama.pl/czy-lekarz-rodzinny-moze-zlecic-badania-krwi-wykrywajace-celiakie.

