# Supplementary material for: Diagnosis, Clinical Presentation and Management of Celiac Disease in Children and Adolescents in Poland
Source: J Clin Med. 2024 Jan 29;13(3):765. doi: 10.3390/jcm13030765 (PMC10856589; doi:10.3390/jcm13030765)
Supplement: Supplementary file 1 [file jcm-13-00765-s001.zip › jcm-2775594-supplementary.pdf]

Supplementary Materials: Diagnosis, Clinical Presentation and Management of Celiac Disease in Children and Adolescents in Poland

The list of supplementary materials:

1. Supplement S1 - sample survey form
2. Table S1. List of symptomatic patients (n = 79, 47 girls), including the division into leading and non-leading symptoms, gender, and CD risk groups.

Coeliac Disease SIG

ESPGHAN
European Society for Paediatric Gastroenterology, Hepatology and Nutrition
CD in Focus

1. Supplement S1 - sample survey form

CELIAC DISEASE MANAGEMENT PRACTICES

**Center ID (assigned regionally)**	
**Patient ID (assigned at center)**	
**Date of birth (YYYY/MM/DD)**	
**Sex**	
**Family history of CD**<br>**(mark accordingly)**	Unknown<br>First degree relative(s)<br>None known so far<br>Other relative(s)
**Was a family member diagnosed before the index patient?**	**Mother –** *Before……………………….Not celiac*<br>**Father -** *Before……………………….Not celiac*<br>**Sister(s) -** *Before……………………….Not celiac*<br>**Brother(s) -** *Before……………………….Not celiac*<br>**Daughter(s) -** *Before……………………….Not celiac*<br>**Son(s) -** *Before……………………….Not celiac*
**When was the diagnosis of CD confirmed?**<br>(Please, enter the date of CD diagnosis regardless of the method by which it was confirmed - with or without intestinal biopsy. Use the date when confirmatory (serology or biopsy) test was performed.)	Date: ______________________________
**Was a patient consuming normal amounts of gluten at the time of diagnosis?**	Yes No
**Were serological tests (laboratory determination of coeliac disease specific antibodies in the serum) performed?**	Yes No
**Why the serological tests were NOT performed?**	- Not available in our practice/institution<br>- Financial reasons (i.e. too expensive)<br>- Rapid TGA test was positive<br>- Villous atrophy was found first, serological tests were not done afterwards<br>- Other tests were being used (e.g. stool tests, bioresonance, food-check, allergy tests)<br>- Patient (parents) refused serological tests<br>- Not recommended by the guidelines<br>- Other (please specify)

**Was diagnosis based on <u>serology only</u> ("no-biopsy" approach)?**	- Yes, diagnosis was based on serology only (single serology sample).<br>- Yes, diagnosis was based on serology only (at least two samples, of which one was the confirmatory one).<br>- No, intestinal biopsy was also performed.
**Was intestinal biopsy performed?**	Yes No
**Why the intestinal biopsy was NOT performed?**<br>If it was not performed.	- Diagnosis was established based on the high titer of coeliac disease specific antibodies (regardless of symptoms) and positive confirmatory serological tests.<br>- Diagnosis was established based only on clear signs of coeliac disease.<br>- Patient (parents) refusal.<br>- Contraindications for biopsy.<br>- Other (please specify).

**Which initial serology tests were (was) performed prior to the diagnosis of CD?**<br>Use the test based on which the decision for a no-biopsy approach was chosen and further diagnostic tests (i.e. confirmatory serological tests) were initiated, regardless whether it was performed in your institution or elsewhere.	
**TGA IgA test**	Positive Negative Not performed<br><br>Specify the result of the TGA IgA test____________<br><br>Date _________________<br>Cut-off value):______________<br>Producer (name of the test):__________________
**TGA IgG test**	Positive Negative Not performed<br><br>Specify the result of the TGA IgA test____________<br><br>Date _________________<br>Cut-off value):______________<br>Producer (name of the test):__________________
**IgA or IgG dGP test (deamidated gliadin peptide Ab)**	Positive Negative Not performed<br><br>Specify the result of the TGA IgA test____________<br><br>Date _________________<br>Cut-off value):______________<br>Producer (name of the test):__________________
**EMA IgA (endomysial Ab) test**	Positive Negative Not performed<br><br>Date _________________
**EMA IgG (endomysial Ab) test**	Positive Negative Not performed<br><br>Date _________________
**IgA or IgG AGA (antigliadin Ab) test**	Positive Negative Not performed
**Rapid TGA test – "point of care" test**	Positive Negative Not performed<br><br>Date _________________
**Was total IgA determined?**	Yes No<br>IgA status:<br>- normal value (or above) for age<br>- lower than normal for age
**Was a second blood sample taken to confirm the positive initial serology? If yes, which test(s) was(were) performed as a confirmatory serological tests? (Mark all tests performed in confirmatory phase)**	None<br>EMA test (date, value, cut-off)<br>TGA test (date, value, cut-off)<br>dGP test (date, value, cut-off)<br>AGA test (date, value, cut-off)<br>Other (please specify)

**When was intestinal biopsy performed?**	Date ____
**Why was intestinal biopsy performed?**	- Because the guidelines require intestinal biopsy to diagnose celiac disease.<br>Because parents demanded the biopsy after the discussion about the possibility for a “no biopsy” approach.<br>- Not eligible for a “no biopsy” approach based on the titer of celiac antibodies.<br>- Not eligible for a “no biopsy” approach because of IgA deficiency.<br>- It is a usual routine in our practice.<br>- Other (please specify)____________
**Was Marsh/Oberhuber classification reported on biopsy specimens?**	Yes No
**Why was Marsh/Oberhuber classification <u>NOT</u> reported on biopsy specimens?**	- Marsh/Oberhuber classification was not possible due to the incorrect orientation or bad specimen quality.<br>- Other classification was used.<br>- Other reasons (please specify)_________
**What was the histological diagnosis? (most severe lesion if patchy)**	Marsh type:_____<br>Other _______
**Were any additional (or second) serological tests performed at the time of diagnosis besides the biopsy?**	Please specify the date, test, its value, cut-off and commercial name of the test.
**Were IgA (IgG, IgM) TGA deposits in the intestinal mucosa determined?**	Positive<br>Negative<br>No, TGA deposit investigation was not performed.
**Were genetic tests (HLA DQ2/DQ8 determination) performed?**	Yes No
**Why were genetic tests not performed?**	- Not available in our practice/institution<br>- Financial reasons (i.e. too expensive…)<br>- Considered as not needed by the current guidelines<br>- Other tests were used<br>- Other (please specify)
**Specify HLA status of the patient.**	DQ2 positive<br>DQ8 positive<br>DQ2 & DQ8 positive<br>DQ2 & DQ8 negative
**Was the patient asymptomatic?**	Yes No

**Diagnostic work-up for coeliac disease in the patient was initiated:** (multiple answers possible)	- Based on symptoms/signs.<br>- Based on screening of risk groups (without any other symptoms/signs indicating possible coeliac disease).<br>- Based on accidentally discovered seropositivity in context of symptoms/signs not attributable to coeliac disease.<br>- Based on population screening.<br>- Based on screening of selected population (e.g. school children…).<br>- Other (please specify)
**Indicate the <u>leading symptom (ONLY ONE)</u> that lead to the suspicion of CD.**<br><br>**Then mark all symptoms/signs present in the patient prior to diagnosis, <u>including the leading symptom</u>.**	Diarrhea<br>Abdominal distension<br>Fatty stool<br>Flatulence<br>Weight loss<br>Growth retardation (in children)<br>Pubertal delay (in children)<br>Loss of apetite (anorexia)<br>Abdominal pain<br>Recurrent vomiting<br>Constipation<br>Dermatitis herpetiformis Duhring (DHD)<br>Dental enamel defects<br>Unexplained fatigue<br>Unexplained irritability<br>Ataxia<br>Headache<br>Iron deficiency with or without anemia
**When were the first symptom(s) (not necessarily the leading symptom) related to CD detected?**	Year (YYYY)<br>Month (MM)<br>(Please make sure to get as reliable information as possible on the onset of first symptoms.)
**When did the patient first visit a specialist in gastroenterology (Paed GI, GI) where the final diagnosis of CD was confirmed?**	Year (YYYY)<br>Month (MM)<br>Day (DD)
**Body weight and height on the date CD was confirmed (biopsy date or confirmatory serology). [kg]**	BM ________ [kg]<br>BH __________ [cm]
**Laboratory findings on the date of diagnosis (or within the three weeks before the diagnosis).**<br>**(Mark whether the outcome of the test was normal, above or below normal.)**	Hb<br><br>MCV<br><br>Ferritin

(if performed)	TSH<br><br>Albumin or total serum protein<br><br>Coagulation (INR)<br><br>ALT<br><br>Bone mineral density
**Does the patient belong to any of the known risk groups?**	Yes No
**To which known risk group does the patient belong? Mark the duration of the underlying risk factor.**	*Not present, <1 year, 1-5 years, 5-10 years, >10 years, since birth, Not known*<br>- Celiac disease in family<br>- IgA deficiency<br>- Thyroiditis<br>- Type 1 diabetes mellitus (TIDM)<br>- AIH (autoimmune hepatitis)<br>- Down syndrome<br>- Turner syndrome<br>- Williams-Beuren syndrome<br>- Other (please specify)________
**Has the patient belonging to a known risk group been serologically tested for CD on a regular basis before the diagnosis?**	Yes No<br>For how many years has he/she been tested?
**How was the final diagnosis FIRST communicated to the patient/family?**	- Face to face visit<br>- Telephone call<br>- E-mail<br>- Written letter<br>- Other (please specify)
**When is the first follow-up clinic visit scheduled after the diagnosis of CD?**	- Follow-up will be done elsewhere<br>- No follow-up visit scheduled<br>- Follow-up will be done _____ months after diagnosis
**Was the patient/family advised to join the local/national coeliac society?**	Yes<br>Unkown<br>No. Please indicate a reason:
**Additional comments (e.g. concomitant disease)?**	

Supplementary Materials: Diagnosis, Clinical Presentation and Management of Celiac Disease in Children and Adolescents in Poland

Table S1. List of symptomatic patients (n=79, 47 girls), including the division into leading and non-leading symptoms, gender and CD risk groups.

		Not CD risk group		CD risk group		Sum
		**leading symptom**	**not leading symptom**	**leading symptom**	**not leading symptom**	
**Abdominal pain**	girls	9	8	4	4	25
	boys	5	2	1	0	8
**Weight loss**	girls	4	5	2	0	11
	boys	4	2	3	1	10
**Growth retardation**	girls	6	7	2	4	19
	boys	8	3	1	1	13
**Diarrhea**	girls	9	3	4	1	17
	boys	4	0	0	0	4
**Abdominal distension**	girls	1	7	2	1	11
	boys	0	1	0	1	2
**Iron deficiency with or without anemia**	girls	2	3	0	1	6
	boys	0	3	1	0	4
**Constipation**	girls	1	5	0	1	7
	boys	0	2	0	1	3
**Dermatitis herpetiformis Duhring (DHD)**	girls	1	0	0	0	1
	boys	2	0	0	0	2
**Unexplained fatigue**	girls	0	1	0	0	1
	boys	1	1	0	0	2
**Unexplained irritability**	girls	0	0	0	0	0

	boys	1	0	0	0	1
**Flatulence**	girls	0	0	0	0	0
	boys	1	0	0	0	1
**Recurrent vomiting**	girls	0	1	0	1	2
	boys	0	0	0	0	0
**Headache**	girls	0	2	0	0	2
	boys	0	2	0	0	2
**Loss of appetite (anorexia)**	girls	0	0	0	0	0
	boys	0	0	0	1	1
**Sum**		59	58	20	18	

Additional information's, based on charts established during OLA and OLAF research for the Polish population:

Percentile grid analysis showed that 12% of patients with CD (58.3% of girls, n=7) had a BMI percentile <5, and all were in the symptomatic group (15.2% of the symptomatic group). Overweight patients BMI percentile >85 accounted for 11% of patients (54.5% girls, n=6), including four obese patients (BMI percentile >95, 3 girls). In the symptomatic group, patients with BMI percentile >85 accounted for 8.9% (n=7, 4 girls), including two girls with BMI percentile >95. 4 patients from the asymptomatic group showed a BMI percentile >85, including 2 patients >95(1 girl).

Weight percentile analysis showed that 19% of patients with CD (36.8% of them were girls, n=7) had a weight <5 percentile, all from the symptomatic group (24.1% of the symptomatic group). Weight >95 percentile was shown only by 4 girls, 2 each from the symptomatic and asymptomatic groups.

Growth percentile analysis showed that 28% of patients with CD (53.6% of them were girls, n=15) had height <5 percentile, including 89.3% (n=25, 15 girls) of this group of symptomatic patients. We found that 3 boys with height <5 percentile were classified as asymptomatic(2 of them with body weight >85 percentile).
